# Through-the-scope clip with anchor prongs for defect closure following myotomy, resection, anti-reflux mucosectomy, fistula management, or bleeding

**DOI:** 10.1055/a-2773-4910

**Published:** 2026-01-27

**Authors:** Jeffrey D. Mosko, Mohammad Al-Haddad, Heiko Pohl, Nikhil A. Kumta, Shannon Melissa Chan, Marvin Ryou, Zaheer Nabi, Ping-Hong Zhou, Haruhiro Inoue, Joyce A. Peetermans, Matthew J. Rousseau, Daniel von Renteln

**Affiliations:** 1 Division of Gastroenterology, Department of Medicine, The Center for Advanced Therapeutic Endoscopy and Endoscopic Oncology, St. Michael’s Hospital, Toronto, Canada; 2 Division of Gastroenterology, Indiana University School of Medicine, Indianapolis, United States; 3 Section of Gastroenterology and Hepatology, Dartmouth Hitchcock Medical Center, Lebanon, New Hampshire, United States; 4 12297 Division of Gastroenterology and Hepatology, NYU Langone Health, New York, United States; 5 71024 Department of Surgery, The Chinese University of Hong Kong, Faculty of Medicine, Hong Kong, Hong Kong; 6 Division of Gastroenterology, Hepatology and Endoscopy, Brigham and Women's Hospital, Boston, United States; 7 78470 Department of Gastroenterology, Asian Institute of Gastroenterology, Hyderabad, India; 8 92323 Endoscopy Center, Zhongshan Hospital Fudan University, Shanghai, China; 9 Digestive Diseases Center, Showa University Koto Toyosu Hospital, Tokyo, Japan; 10 5724 Clinical Endoscopy, Boston Scientific Corporation, Marlborough, United States; 11 Division of Gastroenterology, University of Montreal, Montreal, Canada

**Keywords:** Endoscopy Upper GI Tract, POEM, Motility/achalasia, Non-variceal bleeding

## Abstract

**Background and study aims:**

Through-the-scope endoscopic clips (TTSCs) are essential for defect closure. A newly designed TTSC with anchor prongs can close defects that were challenging with standard TTSCs. We assessed the safety and efficacy of the TTSC with anchor prongs.

**Patients and methods:**

We prospectively studied defect closure using a TTSC with anchor prongs within a multicenter cohort study at 10 sites in six countries. Outcomes were rates of complete defect closure, delayed bleeding, and device- or procedure-related serious adverse events (SAEs).

**Results:**

Fifty lesions among 49 participants were studied. Mean age was 55.6 ±16.6 years, and 24 (49.0%) were male. Indications for closure included endoscopic myotomy procedures (n = 21 lesions, 42.0%), bleeding (n = 9, 18.0%), full-thickness resection (n = 7, 14.0%), submucosal tunneling endoscopic resection (n = 6, 12.0%), endoscopic antireflux mucosectomy (n = 3, 6.0%), perforation or closure of non-bleeding fistula (n = 2, 4.0%), and defect closure after removal of embedded plastic biliary stent (n = 2, 4.0%). Complete defect closure was achieved in 49 lesions (98.0%). No delayed bleeding occurred 30 days after the index procedure. Three patients (6.0%) had four related SAEs: ischemic colitis in a participant with a bleeding colonic fistula (1), submucosal leak in a peroral endoscopic myotomy (POEM) procedure (1), and septic shock and mucosal injury associated with a gastric POEM procedure (1). All related SAEs resolved by 10 days after onset.

**Conclusions:**

The newly designed TTSC with anchor prongs demonstrated safety and efficacy in defect closures after submucosal interventions, with high rates of successful defect closure and no delayed bleeding. (ClinicalTrials.gov number, NCT05653843)

## Introduction

Dissection of submucosal space directly or by creation of a tunnel has been described in various procedures such as to treat upper gastrointestinal submucosal tumors originating from the muscular propria layer, endoscopic full-thickness resection (EFTR) to treat epithelial and subepithelial lesions that cannot be resected using standard polypectomy, endoscopic mucosal resection (EMR) or endoscopic submucosal dissection (ESD), bariatric endoscopic antral myotomy (BEAM) to delay gastric emptying to achieve weight loss, submucosal tunneling endoscopic resection (STER) to treat upper gastrointestinal submucosal tumors originating from the muscular propria layer, peroral endoscopic myotomy (POEM) to treat achalasia, gastric POEM (G-POEM) for gastroparesis, and Zenker POEM (Z-POEM) for Zenker’s diverticulum 1 2 3 . These techniques have shown safe and efficacious endoscopic removal of lesions which, in the past, were primarily resected via open or laparoscopic surgery 4 , and endoscopic management of conditions that were treated with dilation or surgical myotomy 5 . Antireflux mucosectomy (ARMS) involves resection of the gastric cardiac mucosa to reduce the opening of the gastroesophageal junction through healing of the resulting scar, thereby reducing gastroesophageal reflux disease symptoms 6 .

Submucosal endoscopic procedures carry among the highest bleeding risk of any endoscopic procedure, especially in gastric and duodenal submucosal procedures due to the high vascularity of those regions 7 . Postprocedure bleeding rates vary by type of procedure, ranging from 0.2% for POEM 8 to 1.7% for STER to 5.0% for antireflux mucosectomy (ARMS) 9 . Standard through-the-scope clips (TTSCs) are the most common method for closure of the mucosal defect after completion; less often, an over-the-scope closure (OTSC) device or endoscopic suturing may be used 10 . Standard TTSCs are limited by their ability to close large defects and the need for multiple clips. Other closure methods like OTSC devices and endoscopic suturing can be limited by skill requirement or cost considerations. TTSCs with anchor prongs provide the ability to close larger defects because of their ability to anchor and mobilize. This also allows a decrease in the number of clips required.

We studied the efficacy and safety of a newly designed TTSC with anchor prongs (MANTIS clip, Boston Scientific Corporation, Marlborough, Massachusetts, United States) for patients who underwent a wide range of submucosal interventions, full-thickness defect closure, or active bleeding in a multicenter prospective cohort study with 30-day follow-up. Based on published case reports and pilot studies of the study clip 11 12 13 14 15 16 17 , we anticipated a good clinical success rate and adverse events (AEs) comparable to standard TTSCs.

## Patients and methods

### Study design

This was a multicenter, prospective, cohort study of patients who underwent endoscopic clipping using the MANTIS clip (Boston Scientific Corporation, Marlborough, Massachusetts, United States) at 10 sites: four in the United States, two in Canada, and one site each in China, Hong Kong, India, and Japan (ClinicalTrials.gov number, NCT05653843). The MANTIS clip was cleared by the US Food and Drug Administration in August 2022 18 19 . The MANTIS clip is a sterile, radiopaque, single-use endoscopic clip with an 11-mm opening, preloaded on a flexible, rotatable 235-cm-long delivery system and designed to be compatible with forward-viewing endoscopes with working channels ≥ 2.8 mm. It is engineered to enable opening and closing up to five times prior to deployment, with placement following three sequential steps: anchor, mobilization, and closure 20 . MANTIS clips were provided to the participating investigators free of charge for use in this study.

### Patient population

Adult patients were eligible to enroll if they were scheduled for indicated endoscopic clipping per local standard of practice and the attributes of the MANTIS clip were deemed useful for the scheduled procedure. For the current analysis, patients were limited to those who did not have polypectomy, EMR, or ESD (latter data to be published separately).

Patients were excluded if they were enrolled in another investigational study that would interfere with the current study. All centers obtained approval from their respective local ethics committees and all patients provided signed informed consent before nonemergent procedures. In emergent cases (e.g., perforation or intraprocedure acute bleeding) when preprocedure consent was not feasible, consent was obtained after the procedure.

### Study visits

#### Baseline screening

A baseline screening visit included informed consent, eligibility assessment and recording of relevant medical history.

#### Index procedure and postprocedure follow-up

The intervention was placement of the study clip (MANTIS) in the gastrointestinal tract for submucosal endoscopy or resection procedures other than EMR, polypectomy, or ESD. Participants received postprocedure medical care per standard of practice and were subsequently followed for 30 days for assessment of AEs or device events. The last study visit was a telephone interview at 30 days (± 5 days). Subjects were considered lost to follow-up if they failed to return for their scheduled follow-up visits and could not be reached by the study site staff after 3 or more documented attempts.

### Outcomes

Outcomes included: 1) primary efficacy endpoint: complete closure of the defect (defined as no submucosa visible and clips < 1 cm apart); 2) rate of delayed bleeding, defined as a severe bleeding event at the original lesion site that required hospitalization, a blood transfusion (> 5 units), or another invasive intervention (angiographic or surgical) within 30 days after the study clip placement 21 22 ; 3) reinterventions; and 4) rate of serious AEs (SAEs) related to the study clip or the endoscopic portion of the procedure. SAEs were reported on a data extraction checklist with severity and relatedness definitions consistent with the ISO 14155 Standard 23 and MEDDEV 2.7/3 guidance 24 . The level of severity and relatedness of SAEs to the procedure were judged by the site investigators for cases at their own sites.

### Statistical analysis

Baseline characteristics, medical history, outcome measures, and AEs were summarized using mean, median, standard deviation, and range for continuous variables (e.g., age), and proportions for categorical variables. All analyses were performed in SAS version 9.4.

## Results

### Patient and procedure characteristics

Among 49 enrolled participants who had submucosal endoscopy or other procedures, 48 participants had one defect and one participant had two defects (total 50 defects). Mean age was 55.6 years (range 23–80), with most participants being female (n = 25, 51.0%) and Asian (n = 23, 46.9%) or White (n = 22, 44.9%) ( Table 1 ). Six (12.2%) were taking nonsteroidal anti-inflammatory drugs, three (6.1%) were taking anticoagulants, and one (2.0%) was taking antiplatelets at the time of the index procedure.

**Table:** **Table 1** Baseline patient characteristics (N = 49 patients).

Characteristic	Mean ± SD (range) or % (n/N)
**Age, yr** (range)	55.6±16.6 (23.0-80.0)
**Gender: male**	49.0% (24/49)
**Ethnicity**	
Not Hispanic or Latino	2.0% (1/49)
Hispanic or Latino	98.0% (48/49)
**Race**	
Asian	46.9% (23/49)
White	44.9% (22/49)
Black or African American	6.1% (3/49)
Hispanic or Latino	2.0% (1/49)
Medical history*	
No medical history conditions reported	53.1% (26/49)
Coronary artery disease or heart failure	10.2% (5/49)
Gastroesophageal reflux disease	8.2% (4/49)
Gastroparesis	8.2% (4/49)
Abdominal pain, nausea or vomiting	8.2% (4/49)
Bleeding risk	6.1% (3/49)
Diabetes or prediabetes	6.1% (3/49)
Gastrointestinal bleeding requiring intervention	6.1% (3/49)
Hiatal hernia	6.1% (3/49)
Hypertension	6.1% (3/49)
Liver disease/end stage liver disease	6.1% (3/49)
**Actively taking NSAIDs**	12.2% (6/49)
**Actively taking anticoagulants**	6.1% (3/49)
Rivaroxaban	2.0% (1/49)
Warfarin	2.0% (1/49)
Lovenox	2.0% (1/49)
**Actively taking antiplatelets** (clopidogrel)	2.0% (1/49)
*Each patient had one or more of the listed medical conditions; rows are not mutually exclusive. NSAID, nonsteroidal anti-inflammatory drug.	

Indications for clipping included closure after myotomy (n = 21 lesions, 42.0%, including 11 peroral endoscopic myotomies [POEM], 7 G-POEM, 1 Z-POEM, 2 BEAM), bleeding (n = 9, 18.0%), full-thickness resection (n = 7, 14.0%), STER; n = 6, 12.0%), endoscopic antireflux mucosectomy (ARMS; n = 3, 6.0%), perforation or closure of non-bleeding fistula (n = 2, 4.0%), and defect closure after removal of embedded plastic biliary stent (n = 2, 4.0%) ( Table 2 ). In the latter two patients, MANTIS was used to close the mucosal defects after removal of plastic stents embedded on the opposite duodenal wall.

**Table:** **Table 2** Procedure details (N = 50 lesions).

Characteristic	Mean ± SD or median (range) or % (n/N)
**Reason for clipping procedure**	
Myotomy	42.0% (21/50)
Peroral endoscopic myotomy (POEM)	22.0% (11/50)
Gastric POEM (G-POEM)	14.0% (7/50)
Zenker POEM (Z-POEM)	2.0% (1/50)
Bariatric endoscopic antral myotomy	4.0% (2/50)
Bleeding	18.0% (9/50)
Full-thickness resection	14.0% (7/50)
Submucosal tunneling endoscopic resection (STER)	12.0% (6/50)
Endoscopic antireflux mucosectomy (ARMS)	6.0% (3/50)
Perforation or closure of non-bleeding fistula	4.0% (2/50)
Defect closure defect after removal of embedded plastic biliary stent	4.0% (2/50)
**Additional modalities used for closure**	
None	78.0% (39/50)
Injection/sclerotherapy	8.0% (4/50)
Coagulation grasper/forceps	6.0% (3/50)
Hemostatic spray	6.0% (3/50)
Bipolar electrocautery	2.0% (1/50)
Median maximum lesion diameter (range), mm*	20.0 (0.0–40.0)
Median minimum lesion diameter (range), mm*	15.0 (0.0–40.0)
**≥ 30 mm lesions**	18.0% (9/50)
**≥ 20 mm lesions**	66.0% (33/50)
**Mean number of study clips used per procedure (range)**	3.5±1.5 (1.0–6.0)
**Mean number of non-study clips used per procedure (range)†**	1.5±2.3 (0.0–11.0)
**Mean total number of clips used per procedure (range)**	5.0±2.4 (1.0–13.0)
*Zero diameter was recorded for five\5 POEM procedures ^†^ Zero was recorded if no non-study clips were used (28 cases) and other modalities were used to attempt closure.	

Median maximum lesion diameter was 20.0 mm (range 0–40.0) and median minimum lesion diameter was 15.0 mm (range 0–40.0). Thirty-three lesions (66.0%) had maximum diameter ≥ 20 mm and nine (18.0%) had maximum diameter ≥ 30 mm.

For eight lesions (16.0%), one or more of the following additional modalities were used during the index procedure: injection/sclerotherapy (n = 4, 8.0%), coagulation grasper/forceps (n = 3, 6.0%), hemostatic spray (n = 3, 6.0%), and bipolar electrocautery (n = 1, 2.0%) ( Table 2 ). A mean of 3.5 ± 1.5 study clips (range 1–6) and 1.5 ± 2.3 non-study clips (range 0–11) were used per procedure, with mean total number of clips 5.0 ± 2.4 (range 1–13).

### Primary efficacy endpoint: Complete lesion closure

Complete endoscopic clip closure using the Mantis clip after a POEM.Video 1

**Fig. 1:**
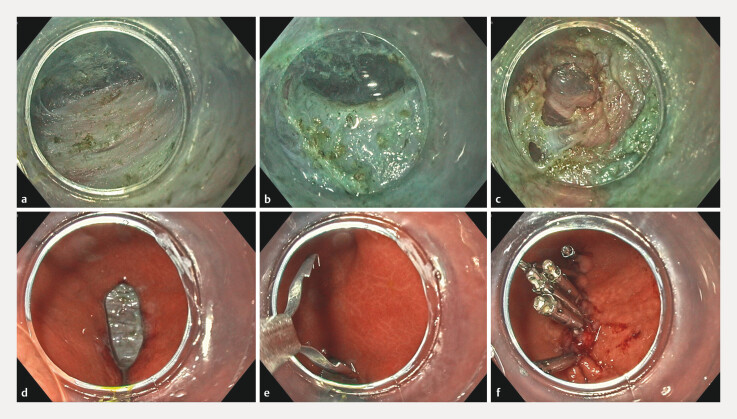
Gastric peroral endoscopic myotomy (G-POEM). **a** Tunnel. **b** Pyloric ring. **c** Myotomy. **d** Mucosal defect. e Mantis clip (open position). **f** Complete endoscopic clip closure.

Among 50 attempted clipping procedures, 49 (98.0%) had complete closure of the defect ( Table 3 , Fig. 1 , Video 1 ). Similar rates of complete closure were seen for lesions ≥ 30 mm (100% [9/9]) and < 30 mm (97.6% [40/41]) ( Table 3 ).

**Table:** **Table 3** Main outcomes (N = 50 lesions in 49 patients).

	Percent (n/N)	95% CI
**Complete closure**	98.0% (49/50 lesions)	89.4%-99.9%
**By lesion size**		
< 30 mm diameter	97.6% (40/41 lesions)	87.1%-99.9%
≥ 30 mm diameter	100% (9/9 lesions)	66.4%-100%
**By procedure type**		
Myotomy	100% (21/21 lesions)	83.9%-99.9%
Defect closure for active bleeding	88.9% (8/9 lesions)	51.8%-99.7%
Full-thickness resection	100% (7/7 lesions)	59.0%-99.9%
Submucosal tunneling endoscopic resection (STER)	100% (6/6 lesions)	54.1%-99.9%
Endoscopic anti-reflux mucosectomy (ARMS)	100 % (3/3 lesions)	29.2%-99.9%
Perforation or closure of non-bleeding fistula	100 % (2/2 lesions)	15.8%-99.9%
Defect closure defect after removal of embedded plastic biliary stent	100 % (2/2 lesions)	15.8%-99.9%
**By endoscopic clip type used**		
Study clip only	96.4% (27/28 lesions)	81.7%-99.9%
Study clip + ≥ 1 non-study clip	100% (22/22 lesions)	84.6%-99.9%
**Delayed bleeding at any site**	0% (0/49 patients)	0%-7.3%

One participant had failure of the primary efficacy endpoint for inability to close a bleeding colonic fistula (described in “Serious adverse events related to the device or procedure” below).

### Rate of delayed bleeding at the original lesion site

No delayed bleeding occurred 30 days after the index procedure in 45 participants with complete follow-up, or in available reports in the remaining four participants.

### Reinterventions

There were two reinterventions in two participants, both of which were upper endoscopy in the inpatient setting. The first reintervention was in a patient with gastroparesis who underwent a G-POEM and developed septic shock on Day 6 after the index procedure. The reintervention took place 1 day after the reported septic shock, and five additional MANTIS clips were placed at the mucosectomy site. Gastrointestinal mucosal injury was also reported at the time of upper endoscopy before study clips were placed. Both SAEs resolved after the procedure with no sequalae. The second reintervention was in a participant with dysphasia who had a POEM procedure, followed by a post procedure barium swallow that revealed contrast extravasation into the submucosal tract created for the POEM procedure. No leak external to the esophageal wall (no perforation) was identified. An upper endoscopy was performed on Day 7 after the index procedure because the initial clip closure was insufficient. Additional study clips were placed and a wound healing agent was applied. The associated SAE was reported resolved 2 days later.

### Serious adverse events related to the device or procedure

Three patients (6.0%) had four SAEs during the study period, three of which were related to the procedure and one of which was related to both the MANTIS clip and procedure ( Table 4 ). The first patient had an attempted closure of a bleeding colonic fistula. Four study clips were placed, but the large fistula could not be closed. No other modalities or other clips were used at the index procedure. The patient developed ischemic colitis on Day 3 which was resolved after 4 days. The patient was treated conservatively with medication and hospitalization but died on Day 19 from worsening sepsis that was not considered related to the study device or procedure. The other SAEs were a submucosal leak in a POEM procedure (1 patient), and septic shock and gastrointestinal mucosal injury associated with a G-POEM procedure (both in 1 patient), both of which resolved by 10 days after onset.

**Table:** **Table 4** Serious adverse events related to the device or endoscopic portion of the procedure (N = 49 patients).

	Number of SAEs	Percent of patients (n/N)	95% CI
Any serious adverse event*	4	6.1% (3/49)	1.3%-16.9%
Septic shock associated with procedure and mucosotomy	1	2.0% (1/49)	0.05%-10.9%
Ischemia colitis ^†^	1	2.0% (1/49)	0.05%-10.9%
Submucosal leak	1	2.0% (1/49)	0.05%-10.9%
Gastrointestinal mucosal injury	1	2.0% (1/49)	0.05%-10.9%
*The septic shock SAE was related to the device and endoscopic portion of the procedure. All other SAEs were related to the procedure only. ^†^ Ischemic colitis occurred associated with defect closure of a bleeding colonic fistula. This case had failure of the primary efficacy endpoint. CI, confidence interval; SAE, serious adverse event.			

## Discussion

In this 30-day prospective study of a newly designed, TTSC with anchor prongs used primarily for endoscopic procedures requiring submucosal intervention, complete closure without delayed bleeding at the original site was achieved in 98% of cases. Two-thirds of the lesions/defects were large (≥ 20 mm), and a mean of 3.5 study clips and 5 total clips were used per procedure.

Seminal animal studies by Sumiyama et al. and Pasricha et al. in 2007 25 26 led to Inoue et al’s first POEM to treat esophageal achalasia in humans in 2010 27 , followed by randomized controlled trials 5 28 29 30 . Our study was conducted at a time when several of these procedures were still in evolution. Innovation and improvement of closure techniques is fundamental to development of these complex procedures, notably in EFTR that requires deeper layer dissection 31 . Our study is notable for high success for closure of large lesions due to the design of the MANTIS clip. The anchor prongs allow for closure of lesions wider than the jaw opening of the clip itself.

We acknowledge strengths and limitations of our study. This is a moderately sized study on closure after advanced techniques including submucosal interventions. Because this was an observational study without a comparator, the current results and cost-effectiveness cannot be compared with other types of TTSCs. In the 44% of cases where both study and non-study clips were used, the added value of MANTIS cannot be quantified. Selection bias was likely, because only cases in which the MANTIS clip was deemed useful were included. We do not have data specifying why MANTIS was chosen; in the setting of a research study, the MANTIS clip might have been used when conventional clips or other techniques could have achieved complete closure. The free clips provided by the sponsor might have created a bias toward increased use of MANTIS during the study. The participating endoscopists were highly experienced and all had prior experience with the MANTIS clip, so their results may be more favorable than expected for most global centers.

## Conclusions

The TTSC with anchor prongs demonstrated efficacy defect closure, with high rates of successful defect closure and no delayed bleeding. The rate of SAEs further supports the clip's safety profile, making it a promising tool for managing endoscopic closures related to submucosal interventions and endoscopic full-thickness resections.

Data Availability Statement

The data, analytic methods, and study materials for this study may be made available to other researchers in accordance with the Boston Scientific Data Sharing Policy ( http://www.bostonscientific.com/en-US/data-sharing-requests.html ).
